# A novel scale‐down cell culture and imaging design for the mechanistic insight of cell colonisation within porous substrate

**DOI:** 10.1111/jmi.12555

**Published:** 2017-03-15

**Authors:** C.M. GABBOTT, Z.X. ZHOU, G.X. HAN, T. SUN

**Affiliations:** ^1^ Centre for Biological Engineering, Department of Chemical Engineering Loughborough University Epinal Way Loughborough UK; ^2^ Department of Materials Loughborough University Epinal Way Loughborough UK; ^3^ Department of Biological Sciences Xi'an JiaoTong‐Liverpool University Suzhou Jiangsu P. R. China

**Keywords:** Cell colonisation, 3D printing, microscopy, porous substrate, scaffold, scale‐down design

## Abstract

At the core of translational challenges in tissue engineering is the mechanistic understanding of the underpinning biological processes and the complex relationships among components at different levels, which is a challenging task due to the limitations of current tissue culture and assessment methodologies. Therefore, we proposed a novel scale‐down strategy to deconstruct complex biomatrices into elementary building blocks, which were resembled by thin modular substrate and then evaluated separately in miniaturised bioreactors using various conventional microscopes. In order to investigate cell colonisation within porous substrate in this proof‐of‐concept study, TEM specimen supporters (10–30 μm thick) with fine controlled open pores (100∼600 μm) were selected as the modular porous substrate and suspended in 3D printed bioreactor systems. Noninvasive imaging of human dermal fibroblasts cultured on these free‐standing substrate using optical microscopes illustrated the complicated dynamic processes used by both individual and coordinated cells to bridge and segment porous structures. Further *in situ* analysis via SEM and TEM provided high‐quality micrographs of cell–cell and cell–scaffold interactions at microscale, depicted cytoskeletal structures in stretched and relaxed areas at nanoscale. Thus this novel scaled‐down design was able to improve our mechanistic understanding of tissue formation not only at single‐ and multiple‐cell levels, but also at micro‐ and nanoscales, which could be difficult to obtain using other methods.

## Lay description

Understanding the complex behaviours of cells cultured within three‐dimensional (3D) biomaterials still remains a fundamental challenge for tissue engineers. It is especially difficult to assess the complex relationships between the cells and the biomaterials at single‐ or multiple‐cell level during tissue culture using common optical microscopes due to their limited focus depth, the relative ‘large’ size and complex structures of 3D biomaterials. To combat this challenge, we proposed a novel scale‐down strategy by ‘deconstructing’ the complex 3D biomaterials into thin sections with basic structures, which will be situated inside miniaturised bioreactors. Thus, the cells cultured on the thin modular substrate can be imaged at single cell level using conventional microscopic techniques. In order to investigate cell colonisation within porous substrate in this proof‐of‐concept study, TEM specimen supporters (10–30 μm thick) with fine controlled open pores (100∼600 μm) were selected as the thin modular porous substrate and suspended in 3D printed bioreactor systems. Human dermal fibroblasts cultured on these modular substrate were imaged through different optical microscopes during cell culture. It was found that complicated dynamic processes utilised by both individual and coordinated cells to bridge and segment porous structures. Further *in situ* analysis via SEM and TEM after cell culture provided high‐quality micrographs of cell–cell and cell–scaffold interactions at microscale, and also illustrated the relation between different mechanical states of the cells and the internal cytoskeletal structures at nanoscale. Thus, this novel scale‐down design was able to improve our mechanistic understanding of cell colonisation within porous biomaterials, which could be difficult to obtain using other cell or tissue culture systems.

## Introduction

Tissue engineering (TE) is defined as an interdisciplinary field that applies the principles of engineering and life sciences toward the manufacturing of biological substitutes to restore, maintain, enhance or replace failing human tissues or organs (Alberti, 2009; Zonari *et al.*, 2012; Gurtner & Chapman, 2016). Since the first report of this research field over 30 years ago (Langer & Vacanti, 1993), dramatic advances and technological developments have been made in TE, thus there is to expect, looking to the near future, the achievement of further intriguing goals (Alberti, 2009; Scarritt *et al.*, 2015). A great expectation, for instance, is to manufacture fully functional tissues or organs to overcome the current limitations related to the shortage of donated tissues or organs, incompatibility problems, and the detrimental effects of long‐term use of immunosuppressive drugs after transplantation (Baiguera *et al.*, 2014). This objective is still hard to achieve due to the following translational challenges. First, even though the recognition and incorporation of the relationships between molecules, cells, tissues and organs as a whole is an inherent characteristic of TE (Scarritt *et al.*, 2015; Gurtner & Chapman, 2016), the mechanistic understanding of these relationships and the underpinning biological processes during tissue formation is very limited (De Kemp *et al.*, 2015). Consequently, as a matter of fact, the current TE approaches are usually administered with the hope that they will do everything nature does without a complete understanding of which biological effects are responsible for the outcomes (Gurtner & Chapman, 2016). Second, a great amount of research efforts have been pursued to develop a huge number of three‐dimensional (3D) biomatrices, media and tissue culture protocols for the manufacturing of specific tissues or organs, while no gold standard or generic procedure has been established (Baiguera *et al.*, 2014; De Kemp *et al.*, 2015). Third, significant scale‐up challenges still exist in TE (Scarritt *et al.*, 2015), as most of the current methodologies for suspension cultures are not applicable for anchorage dependent tissue culture processes. Therefore, new scale‐up strategies based on the mechanistic understanding of the infiltrated cells within heterogeneous and anisotropic 3D scaffolds are urgently needed. Overall, at the core of all these translational challenges is the mechanistic understanding of tissue formation, but thorough investigation of the aforementioned relationships at different levels and the underpinning biological processes of TE based on current tissue culture and analysis methodologies is still a challenging task. First of all, it is almost impossible to distinguish the regulatory functions of each individual architectural and topographic features at nano‐, micro‐ and macroscales, as well as other biochemical and biomechanical properties because of their coexistence in 3D biomatrices (Lin *et al.*, 2014; Yang *et al.*, 2014; Lowenthal & Gerecht, 2016). Monitoring cells infiltrated deep inside 3D scaffolds using conventional optical microscopes is also hardly achievable simply due to their limited focus depth compared with the size of most scaffolds (from mm to cm) (Baradez & Marshall, 2011; Sun *et al.*, 2011). In addressing these problematic issues, an alternative novel scaled‐down study design has been developed in our research group. Briefly, the commonly encountered elementary building blocks of most 3D matrices such as open pores, fibres, curved or flat surfaces with specific structures or topographies at nano‐ and microlevels are identified, and then resembled by a set of thin modular substrate (10–100 μm in thickness) fabricated with microfabrication technologies (e.g. electrospinning, photolithography and 3D printing). The modular substrate are then situated within miniaturised bioreactors, so the cells cultivated on these thin substrate can be imaged noninvasively using common optical microscopes. As this scale‐down design has a complete control of the structural features on the modular substrate as well as the biochemical and biomechanical properties in the miniaturised bioreactors, their regulatory functions on the cultivated cells can be investigated separately. In this proof‐of‐concept (PoC) study, one of the important elementary components of 3D porous matrices, open pore structure, was investigated using this scale‐down strategy. Due to the obvious advantages such as commercially available, low price, suitable thickness (10–30 μm) and most importantly, fine controlled open pore structures with differing shapes and varying sizes, TEM specimen supporters were selected as the thin modular porous substrate, which were suspended in 3D cell culture and imaging systems (3D CCISs) fabricated using 3D printing technology. Human dermal fibroblasts (HDFs) were cultured and imaged noninvasively using optical microscopes during cell culture. *In situ* analysis at micro‐ and nanoscales was also conducted via scanning electron microscope (SEM) and transmission electron microscope (TEM) after cell culture. Thus, the 3D CCISs were employed as a valuable platform to yield mechanistic insights of the aforementioned relationships especially cell–cell and cell–scaffold interactions and the underpinning biological processes during tissue formation at nano‐ and microscales.

## Materials and methods

### Cell culture

Neonatal foreskin human dermal fibroblasts (HDFs, Intercytex, Manchester, UK) were cultured in Dulbecco's modified Eagle's medium (DMEM, Lonza, Slough, UK) containing 4.5 g L^−1^ glucose and supplemented with 10% (v/v) foetal bovine serum (FBS, Fisher Scientific, Loughborough, UK), 2 mM L‐glutamine (Sigma, Dorset, UK), 100 IU mL^−1^ penicillin and 100 μg mL^−1^ streptomycin (Sigma, Dorset, UK), in cell culture T‐flasks at 37°C in a 95% air/5% CO_2_ humidified atmosphere. Media in the flasks were changed twice a week and the cells were continually passaged prior to experimentation at 80–90% confluence using trypsin/EDTA (0.02% w/v solution).

### TEM specimen supporters used as the modular porous substrate

Commercial TEM nickel specimen supporters (diameter: 3.05 mm, thickness: 10–30 μm, bar width: 25–90 μm, Agar Scientific, Stansted, UK) with fine controlled square or hexagonal meshes of different sizes (100, 170, 270, 400 and 600 μm) were utilised as the modular porous substrate in this study. After washed thoroughly using distilled water, dried and autoclaved, the thin modular substrate were either suspended in the 3D CCISs or placed directly on the surfaces of glass coverslips (Agar Scientific) for cell culture experimentations.

### Fabrication of the 3D cell culture and imaging system

**Figure 1:**
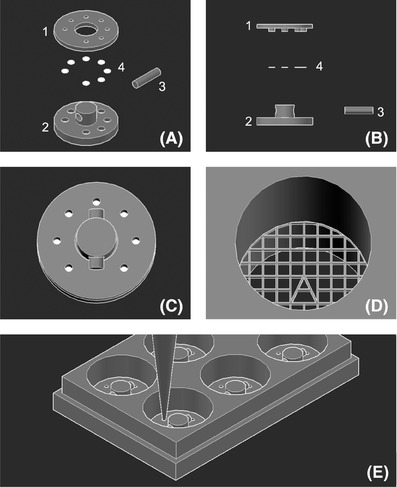
Schematic diagrams of the 3D cell culture & imaging systems (3D CCISs). (A), (B) Nylon 12 was used to 3D print (1) a upper disc with 7 small holes on the edge and a large central hole, (2) a lower disc with 7 corresponding small holes on the edge and a vertical bar with a horizontal socket in the centre and (3) a stopper to accommodate (4) 7 thin modular substrate for each set of 3D CCIS. (C) The upper disc was placed on top of the lower disc through the central hole guided by the vertical bar, and locked in position by the stopper inserted into the socket. (D) The corresponding small holes on both discs were aligned to fabricate 7 small cell culture chambers each with a suspended porous substratum. (E) Each 3D CCIS was assembled, autoclaved and inserted into a well of six‐well plate, an aliquot of 50 μL cell suspension (1×10^5^ cells mL^−1^) was delivered onto each free‐standing substratum via a pipette and incubated at 37°C and 5% CO_2_ for 20–45 min for cell attachment, medium was then added to the plate for cell culture.

Nylon 12 (PA2200, EOS, Warrington, UK) was selected as the 3D printing material, and Selective laser sintering (SLS, Formiga P100, EOS, Warrington, UK) was utilised to print two discs and a stopper for the fabrication of each set of 3D CCIS (Figs. 1A and B). Briefly, on the upper disc (diameter: 30 mm, thickness: 2 mm), 7 small vertical holes (diameter: 3 mm) were created around the edge, while a large central hole (diameter: 11 mm) was also fabricated. In the centre of the lower disc (diameter: 30 mm, thickness: 4 mm), a vertical bar (diameter: 10 mm; height: 7 mm) with a horizontal socket (diameter: 4 mm) was fabricated. Around the edge of the lower disc, 7 corresponding small holes were created, each measuring 2 mm in diameter at the base of the disc, and then expanding to a diameter of 3.5 mm at a height of 0.5–1.0 mm from the base, on which the modular substrate were placed. The upper disc was placed on top of the lower disc through the central hole guided by the vertical bar to make sure all the corresponding small holes on both discs were aligned, thus seven culture chambers each with a free‐standing porous substratum were created (Figs. 1C and D). The stopper (diameter: 4 mm) was then insert into the socket of the vertical bar to lock both discs in position. After washed thoroughly with distilled water, dried and autoclaved, each of the 3D CCISs was inserted into a well of six‐well plate for cell culture (Fig. 1E). In this PoC research, multiple 3D CCISs were fabricated and situated in six‐well plates for multiple comparison experiments.

### Cell culture on free‐standing or surface‐attached modular substrate

An aliquot of 50 μL cell suspension (1 × 10^5^ cells mL^−1^) was delivered directly onto the surface of each free‐standing substratum (Fig. 1E). The six‐well tissue culture plate was then incubated at 37°C and 5% CO_2_ for 20–45 min to allow firm cell attachment onto the porous substrate, medium was then carefully added into each well to submerge the suspended substrate for cell culture. The medium in each well was changed twice week and the cells were imaged noninvasively from underneath the plate using optical microscopes. After cell culture, the 3D CCISs were gently washed with Phosphate‐buffered saline (PBS) and disassembled to gather the thin porous substrate with cultivated cells from the small chambers for *in situ* cell analysis via SEM or TEM.

In the SEM control experiments, glass coverslips (18 mm × 18 mm, Agar Scientific) were inserted into each well of six‐well tissue culture plate, treated with 70% ethanol overnight, washed thoroughly with PBS (×3) and DMEM medium (×1), and then divided into two groups. In the first group, thin modular porous substrate were placed on top of the coverslips prior to seeding cell (1 × 10^4^ cells mL^−1^); while in the second group, cells (1 × 10^4^ cells mL^−1^) were directly seeded on the surfaces of the coverslips. The cultured cells in both groups were monitored using optical microscopes during culture, and analysed *in situ* via SEM after cell culture.

In order to examine the cells cultured on flat surfaces using TEM for comparison purposes, specific TEM specimen supporters (Formvar/Carbon film on nickel 200 mesh, FC200Ni25, EM Resolutions, Saffron Walden, UK) were washed with distilled water, sterilised using 70% ethanol overnight and placed in each well of six‐well plate with the carbon film sides facing upward. An aliquot of 50 μL cell suspension (1 × 10^5^ cells mL^−1^) was delivered onto each carbon film surface via a pipette and incubated at 37°C and 5% CO_2_ for 20–45 min for cell attachment, medium was then added to the plate to submerge the specimen supporters for cell culture. The cells cultivated on the carbon film surfaces were monitored using optical microscopes during culture, and analysed *in situ* via TEM after cell culture.

### Phase contrast microscopy

The cells cultured on glass coverslips, carbon films, suspended or surface‐attached modular porous substrate were monitored and analysed noninvasively using an inverted Phase‐Contrast Microscope (PCM, Nikon Ti, Tokyo, Japan) during culture. Cell orientations and outgrowths were determined from the micrographs using image analysis software (Element, Nikon Ti). Time‐lapse videos of the cells were also captured during cell culture using the same PCM.

### Live cell fluorescent and confocal microscopy

The cells cultured on free‐standing substrate were labelled with Cell Tracker^TM^ Red CMTPX (C34552, Invitrogen, Loughborough, UK) for live cell fluorescent and confocal microscopy during cell culture. An aliquot of 5 μL fluorescent probe Cell Tracker^TM^ reagent stock solution prepared in DMSO was directly added into the cell culture medium to the working concentration (0.5–25 μM). After incubated for 24 h at 37°C and 5% CO_2_, the medium with Cell Tracker^TM^ reagent was replenished with fresh medium. Cell culture was then continued and the labelled cells were imaged and analysed using fluorescent (Nikon Ti) and confocal microscopes (Nikon Cl, Japan) at *λ*_ex_ = 580 nm, *λ*_em_ = 650 nm (for TRITC/Cell Tracker^TM^ visualisation).

### Immune‐fluorescent microscopy

After removed the media from each well of the six‐well plates, the cells cultured on the free‐standing substrate were washed gently with PBS (×3), fixed in IC Fixation Buffer (Fisher Scientific) for 5 min, permeabilised with 0.2% (w/v) Triton X‐100 in PBS for 60 min, and then washed gently with PBS (×3) for 10 min. After being incubated with Phalloidin‐FITC (Sigma, Loughborough, UK, 20 μM) for 1 h, further washed gently with PBS (×3), the fluorescent micrographs of the immune‐labelled cells were captured using fluorescent (Nikon Ti) and confocal microscopes (Nikon Cl, Tokyo, Japan) at *λ*_ex_ = 495 nm, *λ*_em_ = 515 nm (for Phalloidin‐FITC/F‐actin visualisation)

### Scanning electron microscopy

The medium was removed from the six‐well plates after cell culture. The cells cultivated on free‐standing substrate, surface‐attached substrate or coverslips were gently washed with PBS (×3), fixed in IC Fixation Buffer (Fisher Scientific, Loughborough, UK) for 10 min, then gently washed with distilled water, left to dry at room temperature, and coated with gold using a splutter coater (Quorum Q150R S, Laughton, UK). *In situ* analysis of the cells was then conducted via SEM (JSM 7800F, JEOL, Peabody, MA, USA).

### Transmission electron microscopy

After cell culture, the medium was removed from the six‐well plates. The cells cultivated on free‐standing substrate or carbon films were gently washed with PBS (×3), treated with 2% ammonium molybdate solution (Sigma) for 1 min, left dry overnight at room temperature and imaged via TEM (JSM 2000FX, JEOL).

## Results

### Analysis of live cells on free‐standing substrate via optical microscopy

**Figure 2:**
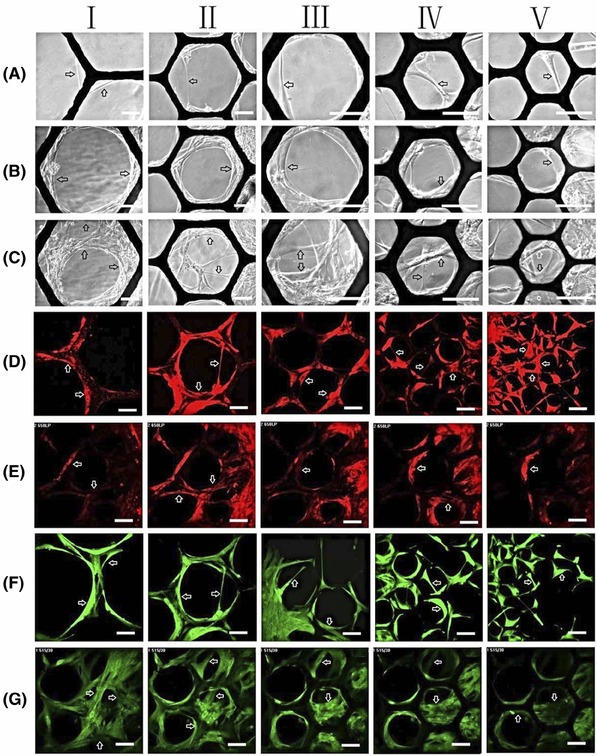
Phase contrast (grey), live‐cell fluorescent (red) and immune‐fluorescent (green) micrographs of human dermal fibroblasts (HDFs) cultured on free‐standing modular substrate with open pores of (I) 600 μm, (II) 400 μm, (III) 270 μm, (IV) 170 μm and (V) 100 μm. After cultured for 2–3 days, (A) individual cells started to bridge and segment small pores or corners with similar sizes (100 and 170 μm). After cultured for more then 3–4 days, (B), (C) more cells joined in and coordinated to bridge and segment large pores. The cells were also stained using (D), (E) Cell Tracker^TM^ (red) and (F), (G) Phalloidin‐FITC (green) for further confocal microscopy. Individual and coordinated cells that bridged and segmented the open pores are pointed by open arrows. Bar = 100 μm.

After seeded onto the suspended thin modular substrate with open pores of varying sizes (100, 170, 270, 400 and 600 μm), the cells were monitored noninvasively with PCM. After cultivated for 2–3 days, most of the HDFs were observed to initially attach and elongate along the struts, and only very few individual cells started to independently bridge either small voids (100 and 170 μm) or corners with similar sizes (Fig. 2A). Analysis of the time‐lapse videos indicated that these individual HDFs usually slid from very small corners towards the void centres, with both terminals of the elongated slender cell bodies attached to either the struts directly or the cells spreading on the substrate. As gradually sliding away from the initial corners, the cells were further stretched and became even slenderer until to the point that the cell bodies were abruptly ruptured. Interestingly, the fractured cell segments were observed to quickly reattach to the struts like live cells. The individual cells were observed to only bridge small voids without reaching the breaking point after 2–3 days’ culture. As culture progressed, more HDFs joined in, they bundled together and behaved as a ‘gigantic multipolar cell’, sliding from small corners towards void centres with multiple attachments to either the struts or the cells spreading on the substrate (Fig. 2B). These ‘gigantic multipolar cells’ were observed to bridge the largest voids investigated (600 μm) after cultured for 6–7 days (Fig. 2C). Thus, all the open pores were divided into smaller voids, which were further bridged and segmented by other individual or bundled cells until the whole porous areas were completely filled with neatly woven cell mats. All the observations indicated that both pore size and culture time period had very significant influences on cell colonisation in the porous substrate, which was confirmed by the repeated cell culture and time lapse experiments (*n* ≥ 3) suggesting the consistency and the reproducibility of the results from the 3D CCISs.

HDFs cultured on free‐standing substrate with open pores of varying sizes (100, 170, 270, 400 and 600 μm) for different time periods were first labelled with Cell Tracker^TM^ Red CMTPX for live cell fluorescent microscopy; then fixed and stained with Phalloidin‐FITC for immune‐fluorescent microscopy. As shown in Figures 2(D)–(G), both live‐cell and immune‐fluorescent microscopies were utilised successfully to capture high‐quality micrographs of the cells cultured in the 3D CCISs. The effects of both pore size and culture time period on cell colonisation, and the strategies applied by the individual (Figs. 2D and F) and coordinated (Figs. 2E and G) cells to bridge, segment and fill in the open pores were all confirmed by these fluorescent micrographs. Optical sectioning of the cell–scaffold composites and 3D volume reconstruction indicated that the membranous cell mats colonised in the porous areas were 5–6 cells or approximately 60 μm thick after the cell cultures were conducted for 1–2 weeks.

### *In situ* analysis of the cells on free‐standing substrate via SEM

**Figure 3:**
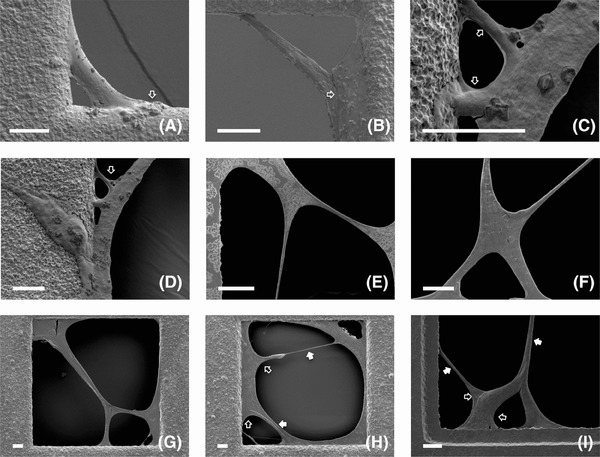
Scanning electron micrographs of human dermal fibroblasts (HDFs) cultured on thin modular substrate with open pores of 170 μm suspended in 3D CCISs. Detailed cell–substrate and cell–cell interactions such as (A) attachment of a sliding cell to the strut through multiple podia; (B) adhesion of a sliding cell to the cell spreading on the substrate; (C), (D) multiple attachments of one terminal of an elongated sliding cell to the strut through thin fibrous structures; (E)–(G) multiple attachments of the multipolar ‘gigantic cells’ to the substrate; (H), (I) stressed (pointed by close arrows) and relaxed areas (pointed by open arrows) in the sliding cells were all illustrated. Scale bar = 10 μm, *n* = 15.

HDFs cultured on suspended thin porous substrate were dried at room temperature, coated and then examined via SEM. The *in situ* SEM micrographs (Fig. 3) illustrated more detailed information about both cell–substrate and cell–cell interactions at microscale with high‐quality and superior precision in comparison to the optical micrographs depicted in previous sections. For example, the initial bridging of very small corners (10–30 μm) by individual cells was clearly illustrated with a very high resolution (Figs. 3A and B). Moreover, very rich details such as the direct attachment of the sliding cell to the strut through multiple podia (Fig. 3A), and adhesion of the sliding cell to the cell spreading on the substrate (Fig. 3B) were clearly depicted. Optical micrographs only demonstrated two attachments between each of the stretched bipolar cells and the struts in the early stage of culture (2–3 days), however multiple attachments through very thin fibrous cell structures (∼1 μm in thickness) were detected by SEM at each terminal of the elongated sliding cells as shown in Figures 3(C) and (D). As culture progressed and more cells joined in, the multiple attachments of the ‘gigantic cells’ and their multipolar morphologies were also displayed (Figs. 3E–G). During the void bridging and segmenting processes, some cells obviously acted as the struts for other cells to slide and bridge, and thus being deformed dramatically due to the mechanical interactions between neighbouring cells, which also caused the formation of visible stressed and relaxed areas in each cell (Figs. 3H and I). Although coordinated as an integrated entity, individual cells within the ‘gigantics’ were still recognisable thanks to the distinct boundaries between neighbouring cells as shown in Figure 3(I).

### SEM analysis of cells on surfaces with/without modular substrate

**Figure 4:**
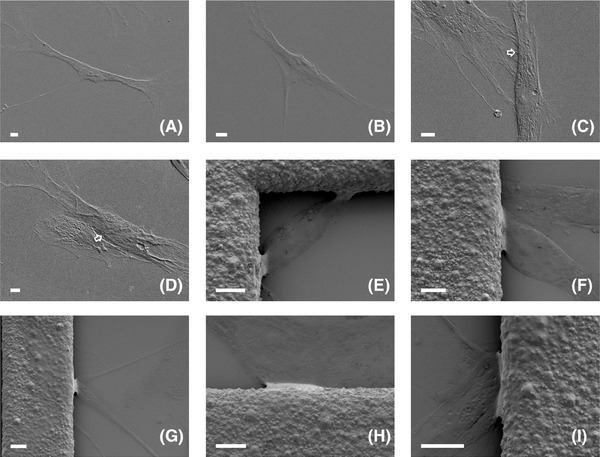
Scanning electron micrographs of human dermal fibroblasts (HDFs) cultured on glass coverslips in the (A)–(D) absence or (E)–(I) presence of thin modular substrate with open pores of 170 μm. The morphologies of (A), (B) individual cells, (C), (D) two encountered cells on glass surfaces and (E)–(I) the cells that attached both to modular substrate and coverslips were depicted. Scale bar = 100 μm, *n* = 3.

HDFs cultured on glass coverslips with or without the presence of thin porous substrate were analysed using SEM. Although the cells on glass surfaces also demonstrated bipolar or multipolar morphologies, the bodies were flattened and more relaxed (Figs. 4A and B) compared to the stretched tubular cell bodies on suspended porous substrate. When HDFs on the glass surfaces made contact with neighbouring cells, they behaved like ‘chance‐encounters’ as no obvious collaborative or coordinated interactions were observed (Figs. 4C and D).

After cultured on the coverslips with the presence of thin porous substrate, only the HDFs that attached to both modular substrate and coverslip surfaces were analysed via SEM. The segments of the cell bodies that bridged the gap between substrate and coverslips demonstrated elongated, stretched but flatted morphologies, while the remaining cell bodies on the coverslips maintained its original flattened and relaxed states (Figs. 4E–I), suggesting the changes of HDF behaviours with the addition of the thin modular substrate on the glass surfaces. A simple thorough rinse experiment using PBS was deliberately conducted when preparing the samples for SEM; and it was found that the modular substrate were hold firmly onto the glass coverslips by the attached cells, indicating the mechanical interactions between the porous substrate and the bridging cells.

### *In situ* analysis of cells on suspended substrate or carbon surfaces via TEM

**Figure 5:**
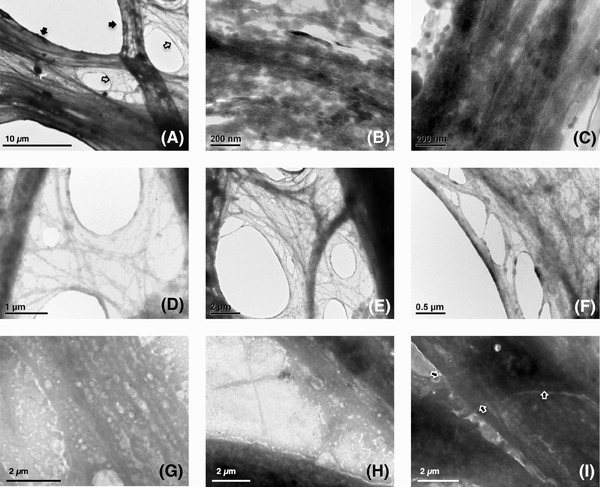
Transmission electron micrographs of human dermal fibroblasts (HDFs) cultured on (A–(F) suspended modular substrate with open pores of 170 μm in 3D CCISs, or (G)–(I) carbon film surfaces. (A) The locations of the stretched dense areas (pointed by close arrows) and the relaxed loose areas (pointed by open arrows); the detailed cytoskeletal structures in the (B), (C) elongated dense areas and (D)–(F) relaxed loose areas of the suspended cells; the detailed cytoskeletal structures in the (G) aligned areas, (H) randomly organised areas and (I) encountered areas (pointed by open arrows) of the cells on carbon films were depicted. (A)–(F) *n* = 15, (G)–(I) *n* = 3.

HDFs cultured on free‐standing modular porous substrate in the 3D CCISs were analysed using TEM and high‐quality *in situ* micrographs of cell anatomy at nanoscale were obtained (Figs. 5A–F) In order to investigate the influences of internal skeletal structure on cell behaviours, cytoskeletons in stretched and relaxed areas of the bridging cells were analysed and compared. As depicted in Figure 5(A), dense and dark structures were identified in the stretched and elongated areas, while the relaxed areas with less dense and more transparent structures were located at the junctions, gaps or the edges of the stretched areas. Further analysis indicated that the microtubules/microfilaments with very high densities were aligned with each other and also bundled tightly to form cable structures in the stretched areas (Figs. 5B and C), while significantly less amount of microtubules/microfilaments were randomly organised and loosely distributed in the relaxed areas (Figs. 5D–F), suggesting the strong correlations between the internal cytoskeletal structures and the mechanical properties of different parts of the cells when bridging and segmenting the void of the porous substrate.

TEM analysis of the cells cultivated on the carbon film surfaces also identified aligned skeletal structures along the direction of cell migration (Fig. 5G) as well as randomly organised microtubules/microfilaments at the junctions or gaps of the aligned cytoskeletons (Fig. 5H). However, the skeletal density of the aligned areas was much lower as compared with the tightly packed structures in the suspended cells. No obvious collaborative or coordinated skeletal interactions but simple overlaps were detected between encountered cells as illustrated in Figure 5(I), which confirmed the SEM analysis of the cells cultivated on glass surfaces.

## Discussion

Due to the intricate characteristics of human tissues such as multiple‐component, heterogeneity and anisotropy, the manufacturing of even ‘simple’ tissues or organs, devoted only to transport functions (e.g. air, food and liquids), represents a challenge, especially in the absence of sufficient mechanistic understandings (Baiguera *et al.*, 2014; De Kemp *et al.*, 2015). Despite considerable advances and technological developments made in TE during the past 30 years, the underpinning biological processes during tissue formation and regeneration are complex, still comprising multiple known, as well as unknown, factors (De Kemp *et al.*, 2015). For example, as one of the three main TE components with critical roles in regulating cells towards the targeted functional outcome (Zonari *et al.*, 2012; Chiron *et al.*, 2012), numerous 3D matrices with multivariate biochemical, biomechanical, drug delivery properties and structural features at nano‐, micro‐ and macroscales have been developed using synthetic or natural polymers (Perán *et al.*, 2012; Shi *et al.*, 2013; Modulevsky *et al.*, 2016). However, our understanding of the extent to which these matrix components contribute to tissue formation when combined with cells, and the attendant opportunities as to what we can do to improve them is still very limited (Gurtner & Chapman, 2016). Therefore, even selecting the suitable scaffold from vast options has always been a challenging task in TE (Ikada, 2006; Place *et al.*, 2009). Since intensive animal testing is problematic due to ethical and economic issues, the majority of 3D biomatrices are initially screened through *in vitro* studies (Wolun‐Cholewa *et al.*, 2013; Wang *et al.*, 2016), and microscopy is one of the technologies commonly utilised to observe the cells infiltrated in the selected matrices. Mechanistic insight is hardly accomplishable using these current tissue culture and assessment methodologies, owing to several problematic issues such as the intricate characteristics of 3D matrices (e.g. co‐existence of multivariate properties, from millimetres to centimetres in size) (Yang *et al.*, 2014; Horner *et al.*, 2016; Olubamiji *et al.*, 2016), the complex interactions of cells with regulatory features at nano‐ and microlevels (Dhandayuthapani *et al.*, 2011; Nuernberger *et al.*, 2011), the limited focus depth and resolution of commonly used optical microscopes (Baradez & Marshall, 2011; Sun *et al.*, 2011). In addressing these challenges, a novel scale‐down strategy has recently developed in our research group by ‘deconstructing’ complex 3D matrices into simple elementary building blocks, which are then ‘resembled’ with a set of microfabricated thin modular substrate, situated in miniaturised bioreactors and evaluated microscopically under fully controlled cell culture conditions. This PoC research aimed to obtain mechanistic insights of cell colonisation within porous substrate using this novel scale‐down design strategy. TEM specimen supporters with fine controlled porous structures were selected as the modular substrate and fabricated in 3D CCISs. Micrographs of the exemplary HDFs cultured on the thin modular substrate were captured at multiple‐ and single‐cell levels using optical microscopes during cell culture; *in situ* analysis was also conducted at micro‐ and nanoscales after cell culture via SEM and TEM with higher resolutions (Clarke, 1973; Vernon‐Parry, 2000). Our research demonstrated that the scale‐down study design is an effective approach to systematically evaluate individual elementary structural components of complex 3D matrices. Thanks to both the thin substrate and the miniaturised 3D CCISs, it was feasible to noninvasively monitor cellular behaviours on the porous substrate using PCM during cell culture. Analysis of phase‐contrast micrographs and time‐lapse videos indicated that HDFs initially attached to the suspended substrate, and only individual cells elongated to bridge small voids (100 and 170 μm) or corners of similar sizes in the first 2–3 days of culture. As culture progressed and cell population increased, more cells joined in and coordinated to cross even the largest gaps investigated (600 μm) after 1 week's culture. These individual and coordinated cells were observed to bridge and divide porous structures into smaller ones, which were then further bridged and divided until the voids were completely filled with a thin layer of neatly weaved cell mats. High‐quality images were captured using fluorescent and confocal microscopy to confirm these observations, but it was still difficult to illustrate further details at micro‐ and even nanoscales such as how the cells changed their morphologies during bridging, and how they attached to substrate or neighbouring cells to coordinate their behaviours. *In situ* SEM analysis at microscale clearly illustrated that multiple podia were utilised to reinforce the attachment of HDFs to the suspended substrate, and they also actively fused cell bodies to coordinate their behaviours when bridging the gaps. Comparison analysis of the cells cultured on coverslip surfaces with the absence or presence of thin modular substrate suggested that HDFs changed their behaviours dramatically due to the addition of the 3rd dimension in suspended substrate. Further TEM analysis at nanoscale provided mechanistic insights into the relationship between cytoskeletal organisation and mechanical state of the cells, that is, aligned parallel dense skeletal structures were always associated with highly elongated stretched cell bodies, while randomly distributed microtubules and or microfilaments were observed in relaxed sections of the cells. A novel control experiment using carbon films made it possible to examine the internal skeletal structures of the cells cultured on smooth surfaces at nanoscales via TEM, and obviously different internal skeletal structures were detected as compared with the cells cultured in free‐standing scaffolds, which confirmed the observations made by SEM. Thus both SEM and TEM not only confirmed and but also provided more detailed mechanistic insights of both individual and highly coordinated multicellular behaviours in suspended porous substrate. This PoC research demonstrated that the scale‐down study design allowed it easy to perform various operations such as system sterilisation, cell seeding, cell culturing and noninvasive cell imaging at single and multiple cell levels using conventional optical microscopes in any normal cell or tissue culture laboratories. Due to the ease of fabrication and operation, multiple 3D SSCIs were set‐up for parallel comparison experiments and consistent results were obtained, suggesting the reproducibility of the results in this research. The utilisation of thin modular substrate also made it possible to directly coat or stain the cells on the substrate for further *in situ* analysis via SEM and TEM. Thus, the 3D CCISs provided a unique, insightful and simplistic platform to investigate the regulatory functions of elementary structures in 3D matrices especially the cell–scaffold and cell–cell interactions at macro‐, micro‐ and nanoscales, which could be difficult to obtain using other cell culture and analysis methods. As the scale‐down design has full control of the cell culture environment, it is possible to evaluate other biochemical, biomechanical properties, and multivariate bioactive molecules (Yang *et al.*, 2014; Lowenthal & Gerecht, 2016) in the presence or absence of specific elementary structures. By culturing different types of cells on the modular substrate, the close spatial relationships and interactions between the cultivated cells, and its influences on the proliferation and migration of each cell type can be also monitored and assessed. Therefore, various structural features, biochemical and biomechanical properties, and even supporter cells that exist in 3D *in vitro* or *in vivo* microenvironments such as stem cell niches can be systematically investigated using this scale‐down design. All these mechanistic insights will be applicable in various areas such as the development of next generation matrices, the formulation of new cell culture media, the production and delivery of functional cells of therapeutic value, and the manufacturing of superior tissue or organs.

## Conclusions

In order to yield mechanistic insights of the cells infiltrated in porous matrices, in this PoC scale‐down study, commercial TEM grids with fine controlled open pores were selected as the modular substrate to resemble the porous structures in 3D scaffolds, and fabricated in miniaturised 3D CCISs. It was demonstrated that this cell culture and imaging system was easy to fabricate and use, and the cells were analysed from multiple‐ and single‐cell levels to micro‐ and nanoscales using conventional optical microscopes, SEM and TEM. Apart from systematic evaluation of the elementary structural features, this novel platform also demonstrated the potential to investigate other biochemical, biomechanical properties and bioactive molecules usually engineered in 3D matrices, which will provide the necessary mechanistic understandings to address the translational challenges in Tissue Engineering.

## Supporting information


**Video 1**: Time lapse video of human dermal fibroblasts cultured on suspended modular nickel substrate with open pores (400 μm).

**Video 2**: Time lapse video of human dermal fibroblasts cultured on suspended modular nickel substrate with open pores (600 μm).

**Video 3**: Volume reconstruction of micro‐graphs of human dermal fibroblasts cultured on suspended modular nickel substrate with open pores (100 μm) and stained with cell tracker (Red), the micro‐graphs were captured through optical sectioning using a confocal microscope.

**Video 4**: Volume reconstruction of micro‐graphs of human dermal fibroblasts cultured on suspended modular nickel substrate with open pores (270 μm) and stained with cell tracker (Red), the micro‐graphs were captured through optical sectioning using a confocal microscope.

**Video 5**: Volume reconstruction of micro‐graphs of human dermal fibroblasts cultured on suspended modular nickel substrate with open pores (400 μm) and stained with cell tracker (Red), the micro‐graphs were captured through optical sectioning using a confocal microscope.
