# Supplementary figures and images for: A novel scale‐down cell culture and imaging design for the mechanistic insight of cell colonisation within porous substrate
Source: J Microsc. 2017 Mar 15;267(2):150–9. doi: 10.1111/jmi.12555 (PMC6849587; doi:10.1111/jmi.12555)

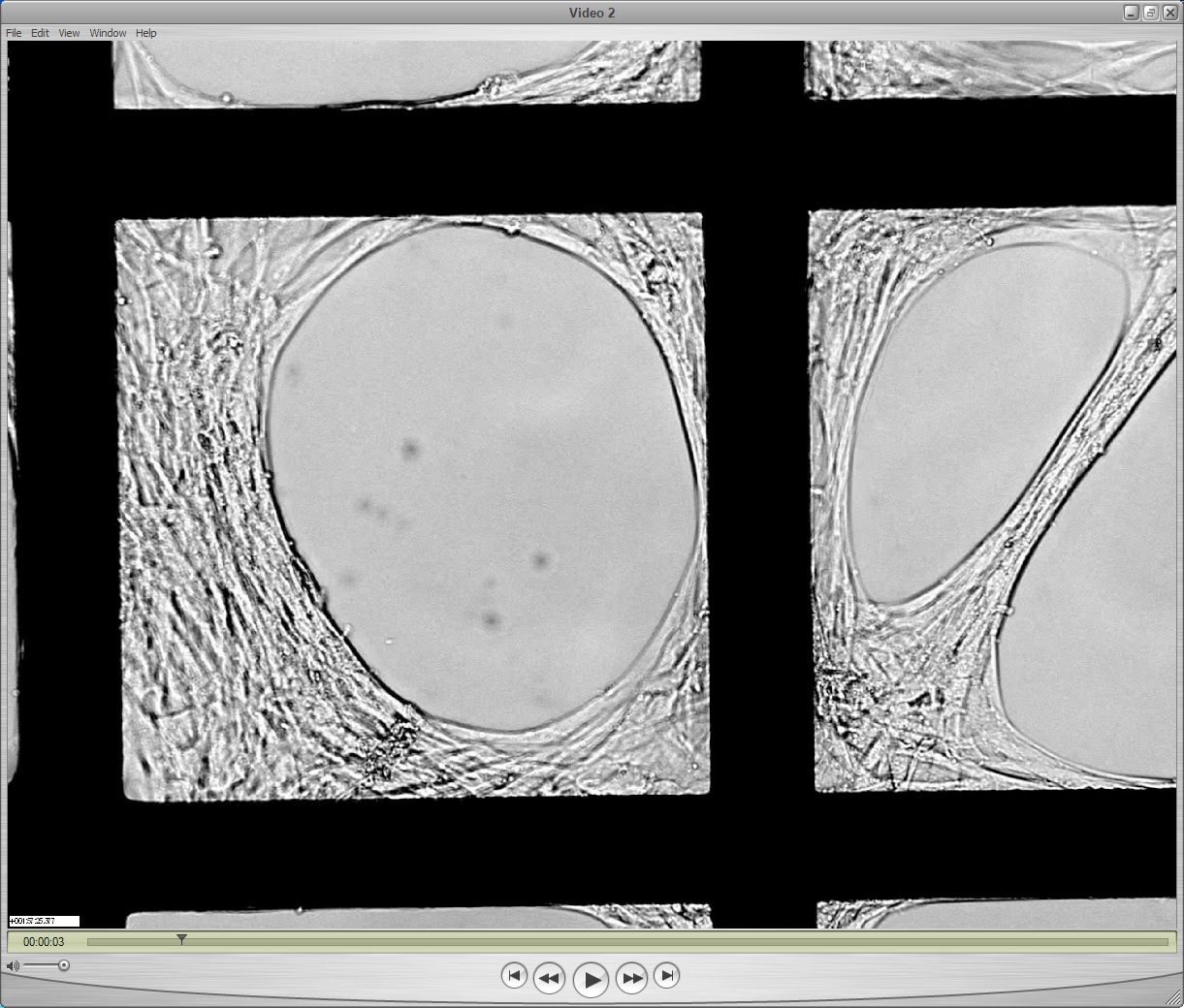

Supplement: Supplementary file 2 — Video 2: Time lapse video of human dermal fibroblasts cultured on suspended modular nickel substrate with open pores (600 μm). [file JMI-267-150-s002.jpg]

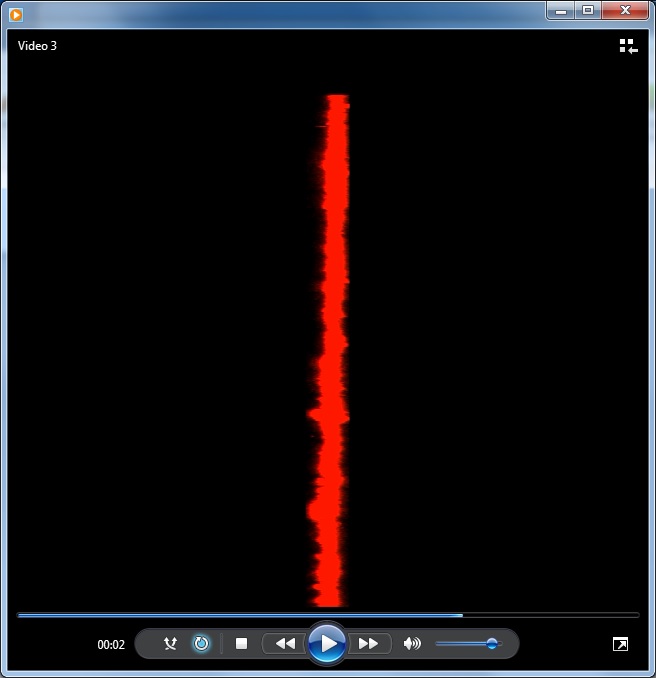

Supplement: Supplementary file 3 — Video 3: Volume reconstruction of micro‐graphs of human dermal fibroblasts cultured on suspended modular nickel substrate with open pores (100 μm) and stained with cell tracker (Red), the micro‐graphs were captured through optical sectioning using a confocal microscope. [file JMI-267-150-s003.jpg]

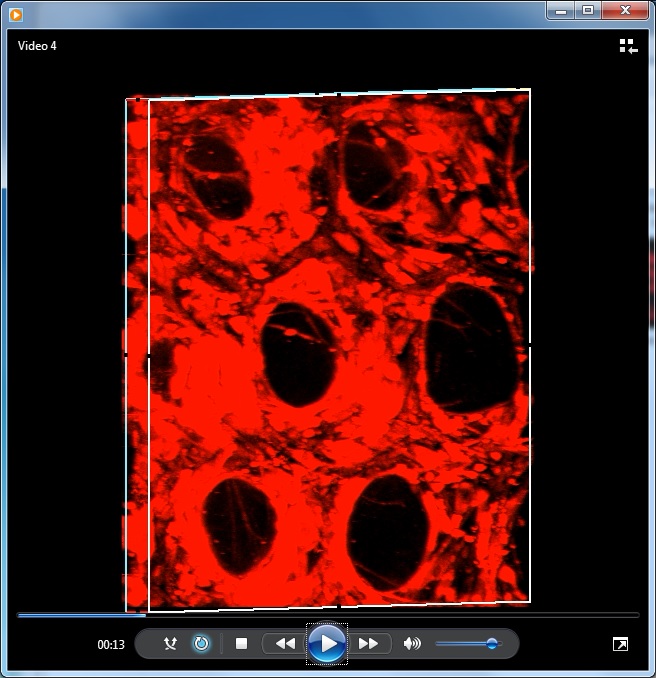

Supplement: Supplementary file 4 — Video 4: Volume reconstruction of micro‐graphs of human dermal fibroblasts cultured on suspended modular nickel substrate with open pores (270 μm) and stained with cell tracker (Red), the micro‐graphs were captured through optical sectioning using a confocal microscope. [file JMI-267-150-s004.jpg]

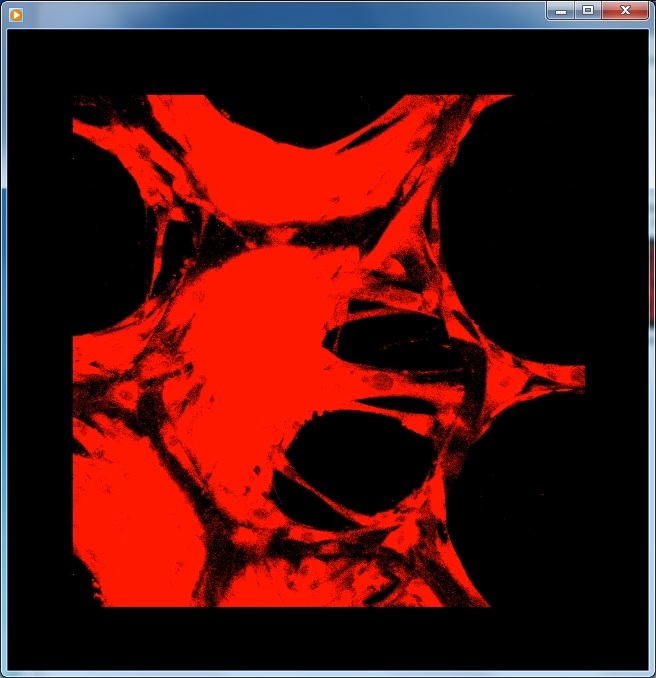

Supplement: Supplementary file 5 — Video 5: Volume reconstruction of micro‐graphs of human dermal fibroblasts cultured on suspended modular nickel substrate with open pores (400 μm) and stained with cell tracker (Red), the micro‐graphs were captured through optical sectioning using a confocal microscope. [file JMI-267-150-s005.jpg]
